# Total free energy analysis of fully hydrated proteins

**DOI:** 10.1002/prot.26411

**Published:** 2022-08-25

**Authors:** Jas Kalayan, Arghya Chakravorty, Jim Warwicker, Richard H. Henchman

**Affiliations:** ^1^ Division of Pharmacy and Optometry, Faculty of Biology, Medicine and Health University of Manchester Manchester UK; ^2^ Department of Chemistry and Biophysics University of Michigan Ann Arbor Michigan USA; ^3^ Manchester Institute of Biotechnology and School of Biological Sciences, Faculty of Biology, Medicine and Health University of Manchester Manchester UK; ^4^ Sydney Medical School, Faculty of Medicine and Health University of Sydney Sydney Australia

**Keywords:** entropy, free energy calculation, multiscale cell correlation, protein hydration, statistical mechanics

## Abstract

The total free energy of a hydrated biomolecule and its corresponding decomposition of energy and entropy provides detailed information about regions of thermodynamic stability or instability. The free energies of four hydrated globular proteins with different net charges are calculated from a molecular dynamics simulation, with the energy coming from the system Hamiltonian and entropy using multiscale cell correlation. Water is found to be most stable around anionic residues, intermediate around cationic and polar residues, and least stable near hydrophobic residues, especially when more buried, with stability displaying moderate entropy‐enthalpy compensation. Conversely, anionic residues in the proteins are energetically destabilized relative to singly solvated amino acids, while trends for other residues are less clear‐cut. Almost all residues lose intraresidue entropy when in the protein, enthalpy changes are negative on average but may be positive or negative, and the resulting overall stability is moderate for some proteins and negligible for others. The free energy of water around single amino acids is found to closely match existing hydrophobicity scales. Regarding the effect of secondary structure, water is slightly more stable around loops, of intermediate stability around β strands and turns, and least stable around helices. An interesting asymmetry observed is that cationic residues stabilize a residue when bonded to its N‐terminal side but destabilize it when on the C‐terminal side, with a weaker reversed trend for anionic residues.

## 1 INTRODUCTION

The structure and stability of proteins are controlled not only by their sequence but also by their solution environment^1^, ^2^, ^3^, ^4^, ^5^, ^6^ comprising salts, solutes, other biomolecules, and numerous water molecules. The influence is mutual, with the properties of these molecules in turn affected by the solvated protein. To understand biomolecular stability and related properties such as folding, binding, and function, it is helpful to be able to quantify and characterize stability in terms of all the constituent molecules of the system, solvent, and solute molecules alike. Stability of a protein with respect to another reference state can be measured experimentally in terms of an equilibrium constant, but this only works if a convenient reference state exists. The absolute stability of a system has no such requirement, depends directly and exclusively on the system itself, and is quantified by the free energy, with lower free energy meaning greater stability. While free energy cannot be measured experimentally, it can be computed from a molecular dynamics (MD) simulation, either from the free energy itself or from energy minus entropy times temperature,^7^, ^8^, ^9^, ^10^, ^11^, ^12^ which can be referred to as energy–entropy (EE) methods. Energy relates to the strength of molecular interactions and entropy relates to molecular flexibility or the probability distribution of configurations.

To calculate total system entropy and energy for biomolecular systems, all‐atom, force‐field‐based simulations are the most useful because they extensively sample the ensemble of configurations over all atoms in the system, unlike coarser‐grain methods, which miss important atomic interactions and degrees of freedom, or electronic‐structure methods, which are much more expensive and still too slow to achieve sufficient sampling. Theoretical approaches to calculate free energy are available for the solvent, such as Poisson–Boltzmann (PB) or Generalized‐Born (GB) continuum solvent methods^13^, ^14^ or the three‐dimensional reference interaction site model, which uses an approximation to the Ornstein–Zernike equation solved on a 3D grid around the protein.^15^, ^16^, ^17^ While these methods avoid the need for ensemble averages over many solvent configurations, they do not yield explicit solvent energy and entropy and nor do they provide the contribution from the protein. The free‐energy method of Meirovitch and coworkers can be applied to both solvent and protein alike,^18^, ^19^, ^20^ but requires a specific Monte Carlo simulation to grow in the molecules as opposed to a standard MD simulation.

Regarding EE methods, energy can be calculated in a straightforward manner from the average system Hamiltonian in a MD simulation. Calculating the entropy is more difficult because it requires determining the probability distribution of quantum states of the whole system, and it is a particular challenge for heterogenous systems comprising both large proteins and large ensembles of small molecules. Of the methods to calculate the entropy of a protein,^21^, ^22^, ^23^, ^24^, ^25^, ^26^ two of the most popular are the multidimensional harmonic approximation, namely normal mode analysis (NMA)^27^, ^28^, ^29^ based on the curvature at the energy minimum and quasiharmonic analysis (QHA)^30^, ^31^, ^32^, ^33^ based on coordinate covariances in MD simulations. Methods using non‐harmonic probability distributions may be more accurate but their correlations are limited to low dimensionality and fail to account for the quantum nature of high‐frequency vibrations in covalently bonded systems. Individual dihedrals^34^, ^35^, ^36^ omit non‐negligible correlations, which can be included either by using distributions along eigenvectors of dihedral covariance,^37^, ^38^, ^39^, ^40^ mutual information expansions,^41^, ^42^ and the minimum spanning tree variant.^43^, ^44^ However, none of these protein–entropy methods are applicable to water because they do not account for translational and rotational entropy which is small for a protein but the main component for collections of small molecules. The solvent contribution can be included using hybrid methods such as molecular mechanics (MM)‐PBSA and MM‐GBSA^13^, ^14^ which combine MM energy with either a PB or GB solvent free energy, a nonpolar surface‐area term, and either NMA or QHA entropy.

Of the entropy methods that are applicable to water around proteins,^45^, ^46^, ^47^, ^48^, ^49^, ^50^, ^51^, ^52^, ^53^ a widely used method is inhomogeneous solvation theory (IST)^45^, ^50^, ^54^, ^55^, ^56^, ^57^, ^58^, ^59^, ^60^, ^61^ based on the solute–solvent density distribution, as well as the closely related Per|Mut method.^62^, ^63^ The two‐phase thermodynamics method, which is based on the velocity autocorrelation function, can be used to calculate water entropy in a wide range of hydration environments.^48^, ^49^, ^52^, ^64^, ^65^, ^66^, ^67^, ^68^, ^69^, ^70^, ^71^ The effect of protein flexibility on hydration can be addressed by considering multiple protein conformations^52^, ^63^, ^72^, ^73^, ^74^, ^75^ possibly supported by conformational clustering,^76^ but this becomes expensive for IST‐type calculations.^77^, ^78^ None of these hydration methods are viable for proteins because having distributions over such a large number of internal degrees of freedom would make them prohibitively expensive.

It would be advantageous to have a single, general method that can account for the free energy of all molecules in hydrated protein systems from an MD simulation. A recently developed EE method that is able to do this is EE‐MCC. This uses the multiscale cell correlation (MCC) method to evaluate entropy of all molecules in the system using data from an equilibrium MD simulation,^79^, ^80^, ^81^ together with energy provided in the usual way from the system Hamiltonian. MCC arose from a multiscale synthesis of the entropy of single flexible molecules using covariance matrices to capture correlations within a molecule^82^ and the entropy of aqueous solutions using cell theory that accounts for multimolecular entropy in a mean‐field manner.^83^, ^84^, ^85^, ^86^ The system is represented as a collection of units of atoms at multiple length scales, with nonbonded units treated in a mean‐field fashion and bonded units as correlated units. The multiscale treatment enables scalability, fast‐convergence and interpretability, which are all desirable features for large systems. The size of the energy well for each unit or collection of units are parameterized in the harmonic approximation from forces, which have been found to have a strongly Gaussian distribution,^80^, ^82^, ^87^ and the quantum harmonic oscillator provides an accurate way to account for entropy in high‐frequency bonded systems. Forces moreover are readily available in a MD simulation and provide an efficient and accurate representation of the average environment of a unit without having to explicitly refer to positions of the surrounding atoms. This is in contrast to coordinate‐based methods which have to define coordinates with respect to external units, which is problematic in continually changing liquid‐phase systems. Entropy is also included for the distribution of different energy wells for each unit, relating to conformations, hydrogen‐bond arrangements, and so forth, which are defined using unit contacts. EE‐MCC has been used to calculate free energy changes in chemical reactions,^88^ octanol–water partition coefficients,^89^ host–guest binding,^90^ and protein–excipient stabilization.^81^ Here, we use EE‐MCC to provide a detailed analysis of the free energy of proteins and their first‐shell water molecules. We examine protein stability, water stability, how they compare with hydrophobicity scales, how they correlate with each other, and whether there is any dependence on various structural features of the proteins.

## 2 METHODS

### 2.1 EE‐MCC method

The Gibbs free energy G is calculated using the EE‐MCC method via the equation G=H−TS, where H is enthalpy, S is entropy, and T is temperature. We explain first the MCC method to calculate entropy of a protein and of water, followed by the calculation of the enthalpy.

### 2.2 MCC protein entropy

In the MCC method, all coordinates are discretized into one or more energy wells, leading to separate terms for the entropy over the different energy wells, termed topographical entropy, and the average entropy within the energy wells, termed vibrational entropy. Moreover, this discretization is done for collections of atoms, termed units, at multiple length scales. For hydrated proteins, the entropy is a sum over the vibrational entropy of units at the polymer (P), monomer (M), and united‐atom (UA) levels, and over the topographical entropy at the UA level of theory in terms of sets of conformational states in each residue^80^ and summarized here. Protein entropy is calculated using

(1) $$S_{\text{prot}}^{\text{total}}=S_{P}^{\text{transvib}}+S_{P}^{\text{rovib}}+S_{M}^{\text{transvib}}+S_{M}^{\text{rovib}}+S_{\mathrm{UA}}^{\text{transvib}}+S_{\mathrm{UA}}^{\text{rovib}}+S_{\mathrm{UA}}^{\text{topo}}$$

where the vibrational entropy relates to the number of states within each energy well and the topographical entropy to the distribution over different energy wells. The polymer corresponds to the whole protein, the monomer to each residue in the protein, and the UA to each heavy atom and all its bonded hydrogens, such as hydroxyl groups, methyl groups, carbonyl carbons and oxygens, and water molecules. The vibrational entropy comprises the translations and rotations of a single or collection of correlated units, labeled as *transvib* and *rovib*, respectively. The vibrational entropy component for each unit or collection of correlated units is calculated from its vibrational frequencies using the equation for a quantum harmonic oscillator

(2) $$S^{\mathrm{vib}}=k_{B}\sum_{i=1}^{N_{\mathrm{vip}}}\left(\frac{{\mathrm{hv}}_{i}/k_{B}T}{e^{{\mathrm{hv}}_{i}/k_{B}T}-1}-\ln\left(1-e^{-{\mathrm{hv}}_{i}/k_{B}T}\right)\right)$$

where $k_{B}$ is Boltzmann's constant, h is Planck's constant, T is temperature, $\nu_{i}$ are vibrational frequencies, and $N_{\mathrm{vib}}$ is the number of vibrational modes for each level. A level is defined as a set of smaller, covalently bonded units: for the polymer level N=1 corresponding to the single protein in the simulation; at the monomer level N is the number of residues in the protein; at the UA level N is the number of UAs in a residue. Vibrational frequencies are calculated for the set of N units from the eigenvalues ($\lambda_{i}$) of separate force and torque covariance matrices using

(3) $$\nu_{i}=\frac{1}{2\pi }\sqrt{\frac{\lambda_{i}}{k_{B}T}}$$

These covariance matrices are constructed from the net forces and torques on each unit derived from the atomic forces outputted from the MD simulation. At each structural level, the matrix elements $\left\langle F_{i}F_{j}/\sqrt{m_{i}m_{j}}\right\rangle$ are mass‐weighted forces for translations and inertia‐weighted torques $\left\langle \tau_{i}\tau_{j}/\sqrt{I_{i}I_{j}}\right\rangle$ for rotations, which are both rotated into the appropriate coordinate system, described below. The force covariance matrices at each length scale have 3N eigenvalues, where N is the number of units. At all but the highest polymer level, the six smallest eigenvalues, which correspond to translation and rotation of the collection of units, are removed to avoid double counting these same degrees of freedom of the single unit they comprise at the higher length scale. For torques, depending on the linearity of each constituent unit, the number of contributed eigenvalues is 3, 2, and 0 for nonlinear, linear, and point constituents, respectively. Concerning coordinate systems, for the polymer, the x,y,z axes are taken as the principal axes with the origin at the center of mass. For residue translations, the same polymer principal axes are used, and for residue rotations a local frame is used: the origin is the average position of the three backbone atoms, and the x,y,z directions of the residue‐level principal axes are defined as the N‐C vector, the vector orthogonal to the NC_*α*_C plane, and the vector orthogonal to both these vectors, respectively. The UA translations use the same local residue axes, and for UA rotation, a more localized frame is used with the origin at the heavy atom and x,y,z axes defined by the average vector of covalent bonds to hydrogens and two axes orthogonal to this.^80^

The last component of protein entropy in Equation (1) is the topographical entropy at the UA level $S_{\mathrm{UA}}^{\text{topo}}$, also called the conformational entropy. As done earlier,^80^ for each dihedral angle comprising four heavy atoms in a residue, the probability distribution is discretized into conformers. Conformers are assigned for each dihedral angle according to the nearest peak in the distribution, where the distribution is constructed with a $30^{\circ }$ bin width, which was found to have sufficient resolution to resolve different conformers. The entropy is calculated from the probability $p_{i}$ of each set of conformers over all dihedrals in the residue using

(4) $$S_{\mathrm{UA}}^{\text{topo}}=-k_{B}\sum_{i=1}^{N_{\text{conf}}}p_{i}\ln p_{i}$$

where $N_{\text{conf}}$ is the number of unique sets.

### 2.3 Water entropy

The entropy of water molecules is divided into vibrational and topographical components at just one structural level

(5) $$S_{W}^{\text{total}}=S_{W}^{\text{transvib}}+S_{W}^{\text{rovib}}+S_{W}^{\text{or}}$$

The vibrational components, $S_{W}^{\text{transvib}}$ and $S_{W}^{\text{rovib}}$, are calculated in the same way as described earlier for protein vibrational entropy at the polymer level because they both correspond to the translation and rotation of a single molecule. The topographical entropy for water here manifests as orientational entropy, $S_{W}^{\text{or}}$, described next.

$S_{W}^{\text{or}}$ accounts for the probability of water molecules accepting and donating hydrogen bonds (HBs) with neighbors and the directional bias of these interactions.^81^ Neighbors are defined based on what solvation shell they are in with respect to the protein surface. A schematic example of neighbor definitions is shown in Supporting Figure S1. The theory builds on previous work for the orientational entropy of flexible liquids^91^ which assumed an isotropic distribution of orientations and previous work on dilute solutions of hydrated ions.^85^ $S_{W}^{\text{or}}$ is calculated as a weighted sum of the logarithm of the effective number of orientations over all observed coordination shells c

(6) $$S_{W}^{\text{or}}=k_{B}\sum_{c}p\left(c\right)\ln\left[{\left(N_{\mathrm{eff}}\pi \right)}^{\frac{3}{2}}p\left({\mathrm{HB}}_{\mathrm{av}}\right)/\sigma \right]$$

where σ=2 is the symmetry number of water and $N_{\mathrm{eff}}$ is the effective number of available neighbors that can be hydrogen‐bonded to without bias, and $p\left({\mathrm{HB}}_{\mathrm{av}}\right)$ expresses the probability that neighboring molecules are in the correct orientation for the molecule of interest to form its HBs. In the homogeneous water case,^91^ $p\left({\mathrm{HB}}_{\mathrm{av}}\right)=0.25$. More generally, it is calculated as a weighted average of the probability of forming HBs to each type of neighbor n

(7) $$p\left({\mathrm{HB}}_{\mathrm{av}}\right)=\frac{\sum_{n}p\left({\mathrm{HB}}_{n}\right)N_{n}}{N_{c}}$$

where $N_{n}$ is the number of neighbors of type n, $N_{c}$ is the total number of neighbors in the coordination shell such that $N_{c}=\sum_{n}N_{n}$, and $p\left({\mathrm{HB}}_{n}\right)$ is the probability for a water molecule to donate or to accept from a neighbor of type n, calculated as

(8) $$p\left({\mathrm{HB}}_{n}\right)=\frac{p\left(D_{n}\right)}{p\left(D_{n}\right)+p\left(A_{n}\right)}\times \frac{p\left(A_{n}\right)}{p\left(D_{n}\right)+p\left(A_{n}\right)}$$

where $p\left(D_{n}\right)$ and $p\left(A_{n}\right)$ are the probabilities of donating to or accepting from a neighbor of type n, respectively. Thus $p\left(D_{n}\right)=N_{D_{n}}/\sum_{n}N_{D_{n}}$, where $N_{D_{n}}$ is the number of donations to neighbor n and similarly $p\left(A_{n}\right)=N_{A_{n}}/\sum_{n}N_{A_{n}}$. The effective number of neighbors available to form HBs to in Equation (6) takes into account how often the neighbor is involved in a HB. It is calculated using

(9) $$N_{\mathrm{eff}}=\sum_{n}\frac{p\left({\mathrm{HB}}_{n}\right)N_{n}}{0.25}$$

where 0.25 is the ideal number for two HBs with 0.5 probability to accept and donate. HBs are defined topologically^84^, ^85^, ^92^ between a hydrogen and the most favorably interacting acceptor, namely the acceptor for which $q_{D}q_{A}/r^{2}$ is most negative, where $q_{D}$ and $q_{A}$ are the charges of the donor and acceptor atoms, respectively, and r is the distance between them.

### 2.4 Protein hydration shell

**FIGURE 1:**
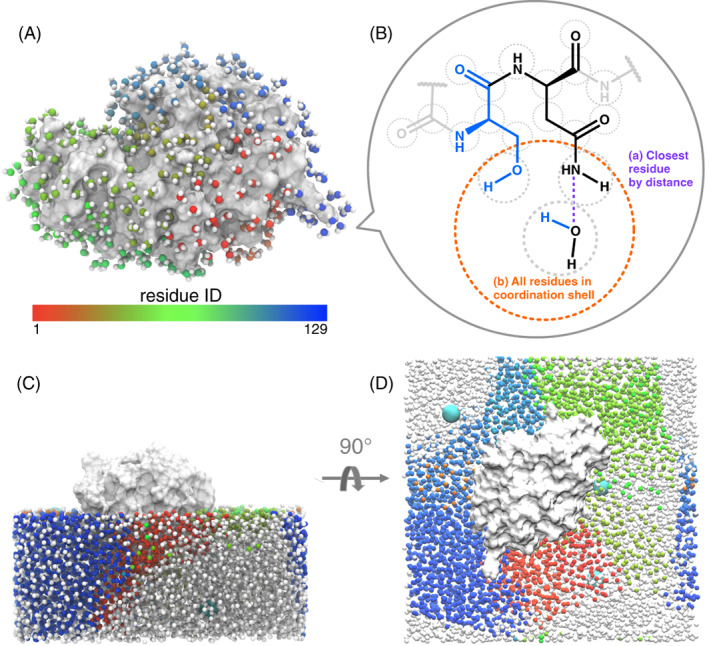
(A) Waters in the first coordination shell of lysozyme, colored by their closest residue united atom (continuously shaded from red to green to blue to represent residue IDs in the protein sequence). (B) Water molecules grouped by (A) the closest residue (dashed purple line) in its coordination shell (dashed orange circle) and (B) all residues pairs in the coordination shell. United atoms are marked by dashed gray circles. (C,D) Two orientations of the lysozyme protein (white) and half the water, residue IDs colored as in part (A). Waters nearest to ions are colored white

To calculate the free energy of hydrated proteins, we only consider water molecules in the coordination shell of the protein as depicted in Figure 1(A). Although longer‐range effects may be present, causing water molecules to be perturbed beyond the protein surface,^68^, ^93^, ^94^ they are weak and difficult to account for because of statistical noise involving a large number of molecules. To analyze the different types of water molecules, we group them according to the nearest non‐water UA (Figure 1(B)), whether in the protein or an ion, and the nearest residue, defined as the residue that contains the nearest UA. This is similar to the so‐called proximity criterion,^95^, ^96^ which groups waters based on the distance to their closest solute atom, as depicted in Figure 1(C,D). As well as considering a hydration water's closest residue, we also consider its nearest two residues when a second such residue exist. United‐atoms in a molecule are defined to lie in the coordination shell of another UA if they are not blocked by a closer UA using the relative angular distance (RAD) algorithm.

(10) $$\frac{1}{r_{\mathrm{ij}}^{2}}>\frac{1}{r_{\mathrm{ik}}^{2}}\cos\theta_{\mathrm{jik}}$$

where $r_{\mathrm{ik}}$ and $r_{\mathrm{ij}}$ are distances of particle i from particles k and j, respectively, $\theta_{\mathrm{jik}}$ is the angle between k and j formed at i. The RAD method is applied at the UA level, which is defined as a heavy atom and its bonded hydrogen atoms, and the center point of a UA is at the heavy atom. We also consider the stability of water according to protein secondary structure, grouping water molecules according to their nearest secondary structure elements as defined by STRIDE.^97^

### 2.5 Calculation and partition of the enthalpy

The enthalpy H of the whole system is directly accessible in an equilibrium MD simulation as the ensemble average of the system Hamiltonian as specified by the force field plus a pressure‐volume term PV. The total PV term at ambient pressure is on the order of tens of kJ mol^−1^ but effectively the same on a per‐water or per‐residue basis, on the order of 2 J mol^−1^ for water and 10 J mol^−1^ for residues, and so is ignored in this analysis. Consequently, in this work, we use the terms enthalpy and energy interchangeably. H can be partitioned into atomic terms $U_{i}$ which equals the sum of all energy terms in which that atom participates, be it bonded or nonbonded, divided up equally between contributing atoms. The enthalpy of any unit j, whether polymer, monomer, or UA, is calculated as a sum over all constituent atoms using

(11) $$H_{j}=\sum_{i}^{N_{j}}\left(K_{i}+U_{i}\right)$$

where $K_{i}$ is the atom's kinetic energy and $N_{j}$ is the number of atoms in the unit.

### 2.6 Simulation protocol

All systems are generated and minimized with Assisted Model Building and Energy Refinement (AMBER) 18^98^ and then subject to MD simulations in Large‐Scale Atomic/Molecular Massive Parallel Simulator (LAMMPS),^99^ which we make use of because it outputs atom‐specific energies. Simulations are performed of each of the four proteins lysozyme, α‐chymotrypsin, α‐lactalbumin, and ribonuclease Sa. The protein structures are taken from the Protein Databank using the IDs given in Table 1 which also lists the number of residues $N_{R}$ and UAs $N_{\mathrm{UA}}$ in each protein.^100^

**TABLE 1:** PDB ID, net charge, and numbers of residues and united atoms for the proteins

Protein	PDB ID	Q	$N_{R}$	$N_{\mathrm{UA}}$
Lysozyme	2vb1	+8	129	1001
α‐Chymotrypsin	1yph	+3	241	1751
α‐Lactalbumin	1f6r	−6	123	993
Ribonuclease Sa	1rgg	−7	96	746

Protonation states of titratable groups in the protein are set appropriate to a pH of 7 using the PDB2PQR online server,^101^ with the only nonstandard protonation of Asp88 in α‐lactalbumin. For histidine, the neutral HIE tautomers are used. The resulting net protein charges Q are listed in Table 1. Each system is constructed in a cubic box by solvating with water and either sufficient Na^+^ or Cl^−^ ions using Packmol to neutralize the system,^102^ with 20 Å of water around each protein. MD simulations of each of the 20 amino acids in water are also run to examine their solvation and provide reference data to compare with residues in the protein. Each simulation contains one amino acid, which is acetyl‐capped at the N‐terminus and methyl‐amide‐capped at the C‐terminus, and 900 water molecules. Each amino‐acid system and box of 900 water molecules are run in triplicate to provide an estimate of the errors involved. Each system is constructed in a cubic box by solvating with water out to 10 Å using Packmol. The four proteins and the single capped amino acids are modeled using the Amber FF14SB force field,^103^ TIP3P for water,^104^ and the Joung and Cheatham TIP3P parameters^105^ for the Na^+^ and Cl^−^ ions. System topology files are generated using AMBER 18 and then minimized for 5000 steps using steepest descents. The files are converted into LAMMPS formatted input files using InterMol.^106^ Using LAMMPS, temperature is slowly increased to 298 K under constant NVT (number volume temperature) for 0.2 ns followed by number pressure temperature (NPT) equilibration for 5 ns under NPT conditions with a 1 fs time step. Production simulations are run for 50 ns under the same NPT conditions and a 2 fs time‐step. Temperature and pressure are controlled using a Nose–Hoover thermostat and barostat, respectively. Temperature is relaxed every 0.2 ps and pressure is relaxed every 0.5 ps with an isotropic stress tensor across box dimensions. Nonbonded interactions are cut off at 9 Å, and long‐range interactions beyond this distance are calculated with particle–particle particle–mesh.^107^ The SHAKE algorithm is used to constrain all bonds and angles to hydrogen atoms.^108^ The pe/atom and ke/atom flags in LAMMPS are set to output trajectories of potential and kinetic energies per atom for molecular energy analysis. Force and coordinate trajectories are outputted for the entropy analysis. Trajectories are saved every 10 ps to give 5000 frames for analysis for each simulation run. Previous work on proteins has shown that this sampling protocol is sufficient to obtain converged entropies.^80^ Output files are read in using the MDAnalysis python library.^109^ The energy and entropy of water and protein energy are analyzed using an in‐house python program POSEIDON Beta V2.0 available at https://github.com/jkalayan/PoseidonBeta. Protein entropy is analyzed using earlier software CodeEntropy^80^ which is available at https://github.com/arghya90/CodeEntropy. This requires converting the LAMMPS topology and trajectory files to PSF and DCD formats using CPPTRAJ.^110^

### 2.7 Analysis of structure–thermodynamics correlations

To assess the trends in energy and entropy in the protein simulations, several other quantities are calculated on a per‐residue basis:

1. HR: Hydrophobicity of each type of amino acid ranked by the calculated free energy of water molecules around amino‐acid side chains, where HR value increases with hydrophobicity.
2. RMSD: Average root‐mean‐square deviation of side‐chain heavy‐atoms on each residue aligned and compared to the simulation starting structure using CPPTRAJ.^110^
3. $N_{\mathrm{Wc}}$: Average number of water molecules in the coordination shell of the closest residue.
4. $N_{\mathrm{Rc}}$: Average number of residue UAs in the coordination shell of a residue.

Correlations between protein structural features and thermodynamic properties of residues and water molecules are determined using a covariance matrix with elements

(12) $$\mathrm{cov}\left(X,Y\right)=\frac{1}{N}\sum_{i=1}^{N}\left(X_{i}-\bar{X}\right)\left(Y_{i}-\bar{Y}\right)$$

where for each value $X_{i}$ in feature X containing a total of N values, the deviation from the mean $\bar{X}$ is assessed simultaneously with the deviation from the mean of values in feature Y. Each value is normalized so that the mean is zero and standard deviation σ is one. This gives matrix elements between 1 (fully correlated covariance) and −1 (fully anticorrelated covariance), where 0 is no correlation in the covariance of two features.

## 3 RESULTS

### 3.1 Free energy of water around proteins

The free energy, energy, and entropy per water molecule for water in the coordination shell of each protein and in bulk are shown in Table 2, with superscript “vib” encapsulating both “transvib” and “rovib” in Equation (1).

**TABLE 2:** Water entropy components, enthalpy, free energy, and count in the protein coordination shell

	$S_{W}^{\mathrm{vib}}$	$S_{W}^{\text{or}}$	$S_{W}^{\text{total}}$	$H_{W}$	$G_{W}$	$N_{\mathrm{Wcp}}$
	($J\;K^{-1}\;{\mathrm{mol}}^{-1}$)			($\mathrm{kJ}\;{\mathrm{mol}}^{-1}$)		
Lysozyme	67.2	6.8	74.0	−33.2	−55.3	464
α‐Chymotrypsin	67.1	6.5	73.7	−33.3	−55.2	739
α‐Lactalbumin	66.9	6.6	73.4	−33.5	−55.4	477
Ribonuclease Sa	67.2	6.4	73.5	−33.4	−55.4	396
Bulk water	68.1	10.7	78.8	−32.3	−55.8	‐

Per‐water errors in enthalpy were calculated from their standard deviations and found to be in the range 0.01–0.02 kJ mol^−1^. With errors in entropy having been found elsewhere to be slightly smaller,^88^ this means that the numbers shown are accurate to the precision used. The total number of water molecules in the protein coordination shell $N_{\mathrm{Wcp}}$ in Table 2 is all water molecules that have a residue UA in their coordination shell. Consistent with what has been seen elsewhere for hydration,^111^, ^112^, ^113^ there is mild EE compensation across all proteins, the highest entropy and enthalpy being for lysozyme and the lowest for α‐lactalbumin. This trend could be related to the charge of the proteins, given that the entropy and energy per water molecule are lower near the two negatively charged proteins, α‐lactalbumin and ribonuclease Sa (net charges in Table 1). Moreover, both these proteins have overall free energies that are slightly more stable, that is to say, more negative, because negatively charged residues have fewer but stronger HBs because of the local deficiency of donors. This is consistent with the preferred solvation of anions over cations in water due to water's asymmetric structure,^85^, ^114^, ^115^, ^116^ making it is easier for a water molecule to donate to two strong HBs with its well‐spaced hydrogens than it is to accept from two hydrogens via its single oxygen. The trend in $N_{\mathrm{Wc}}$ reflects the size of the protein: the larger the protein, the more first‐shell water molecules there are around the protein. Note that the values of H and S for bulk TIP3P are higher than the experimental values of −34.1 kJ mol^−1^ and 69.9 J K mol^−1^ as noted elsewhere,^117^, ^118^ with the orientational entropy in this work being even higher and in better agreement with other methods^118^ compared to the earlier value using the tetrahedral model.^117^

**FIGURE 2:**
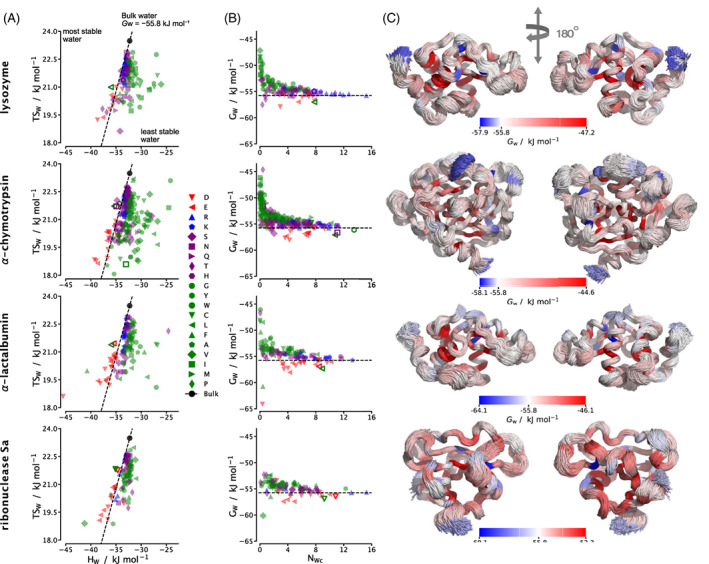
(A) Energy versus entropy for water around each residue in each protein. Dashed lines correspond to hypothetical values of enthalpy and entropy that give the free energy of bulk water ($-55.8\;\mathrm{kJ}\;{\mathrm{mol}}^{-1}$) and the black circle is the actual computed bulk‐water value. (B) Water free energy versus number of water molecules closest to a particular protein residue. Negative residues are red, positive residues are blue, polar residues are purple, and nonpolar residues are green. Terminal residues are represented as open markers. (C) Superimposed protein structures of 2500 frames from 50 ns trajectories shown from the front and back (left and right). Each residue is colored by the free energy of water molecules closest to that residue relative to bulk water (blue more stable and red less stable)

Water molecules around each of the four proteins are further assessed in Figure 2 based on their closest residue. Again, the EE compensation of water molecules is clearly present. Hypothetical energy and entropy values required to give the bulk water free energy of $-55.8\;\text{kJ}\;{\text{mol}}^{-1}$ are shown as a dashed line in Figure 2(A). Molecules to the left of this line are stabilized and those to the right are destabilized. We first note that there is much more variation in energy than in entropy. Generally, water around negative residues are more stable than bulk water, while water molecules around positive and polar residues are similarly stable or slightly less stable. These kinds of water molecules show little dependence on the number of water molecules in their shell, which relates to the degree of burial. Water around nonpolar residues are the most diverse and scattered in entropy and energy because of the contribution of orientational entropy which depends in a more multi‐body fashion on the anisotropy and size of the coordination shell, rather than solely on the strength of interactions. Water molecules that have lower $N_{\mathrm{Wc}}$ are most likely more buried in the protein. They are typically near hydrophobic residues and have a higher free energy. The burial effect may be examined in more detail from the free energy, enthalpy, entropy, and entropy components versus the number of surrounding protein UAs $N_{\mathrm{UAc}}$ (Supporting Figure S2). The two main trends are that orientational entropy decreases strongly with the degree of burial, likely due to asymmetry and confinement, and that the spreads of the other terms increase for more buried waters, going from strongly bulk‐like to either higher or lower, again with the majority of waters less stable than in bulk. The variation in nonpolar water free energy in Figure 2(A) is different according to the proteins, with ribonuclease Sa having less scatter than the other proteins. This appears to correlate with the size of the protein, which also relates to the number of nonpolar and buried residues, with α‐chymotrypsin having the most and ribonuclease‐Sa the least. The location of each water around the flexible protein surface (Figure 2(C)) does not display any notable trends, except that water molecules are seen to have higher free energy in more buried regions, as noted earlier.

### 3.2 Free energy of water contacting two protein residues

**FIGURE 3:**
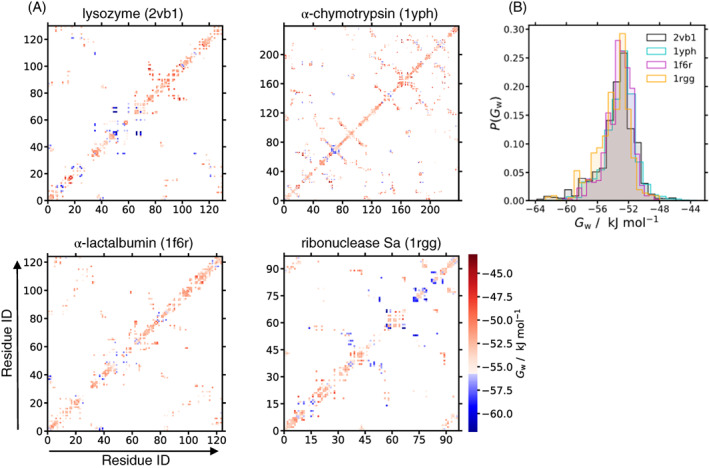
(A) Free energy $G_{W}$ of water in contact with pairs of protein residues colored in red for less stable and blue for more stable than bulk water. (B) Normalized probability histograms of $G_{W}$ around each protein

To understand how the free energy of water is affected by being near multiple residues, plotted in Figure 3(A) is the free energy of water molecules that are in contact with the UAs of two or more residues for the four proteins. This plot also reveals which residues are close enough to interact with the same water molecule and whether the amino acids are adjacent or distant in sequence, the latter being more likely in a binding site. Equivalent plots in terms of enthalpy and entropy are provided in Supporting Figure S3. The trends are found to be fairly similar to those for the nearest single residues. For example, the active site of lysozyme^119^ (Glu35 and Asp52) is surrounded by more stable water molecules than bulk, which is partly because of the negatively charged residues. Cation‐binding sites also generally display stabilized water. For α‐lactalbumin, some stabilized water molecules are found in calcium‐binding sites, one site being at residues Asp82, Asp87, and Asp88, and the backbone carbonyls of residues Lys79, Asp4, and Asp8, and the other site at Thr38, Gln39, Asp83, and the backbone carbonyl of Leu81,^120^ with the water molecules in the more exposed second binding site being more stabilized. Some zinc‐binding sites are also surrounded by stabilized water molecules and again generally appear at more solvent‐exposed regions. On the other hand, water molecules in some nonpolar regions are destabilized and would therefore be easier to displace by nonpolar molecules. The histograms of $G_{W}$ values (Figure 3(B)) are similar for each protein, but ribonuclease‐Sa has a greater number of stabilized waters, consistent with it having the most negative charge, as noted earlier.

### 3.3 Free energy of water according to protein secondary structure

Table 3 contains the values of ${\mathrm{TS}}_{W}$, $H_{W}$, and $G_{W}$ around each type of secondary structure averaged over all four proteins, together with the number percentage of residues and water molecules associated with each type.

**TABLE 3:** Water entropy, enthalpy, free energy, and residue and water percentages for protein secondary structure

Secondary	${\mathrm{TS}}_{W}$	$H_{W}$	$G_{W}$	% Residues	% Water
Structure	($J\;K^{-1}{\mathrm{mol}}^{-1}$)	($\mathrm{kJ}\;{\mathrm{mol}}^{-1}$)			
Bridge	20.1	−34.0	−54.1	3	3
Coil	20.2	−33.9	−54.1	17	20
Extended	20.3	−33.5	−53.8	19	12
Turn	20.4	−33.4	−53.8	18	17
3_10_ Helix	20.3	−32.9	−53.2	4	4
α Helix	20.4	−32.9	−53.3	18	17

It can be seen that $G_{W}$ has a ranking from most to least stable of bridge, coil < extended, turn < 3_1__0_ helix, α helix. Most of this trend is governed by a less negative enthalpy that is slightly offset by a larger entropy. Evidently, water appears to be less stable around the more ordered helices and sheets and is more stable around the less ordered coils and isolated bridges. This is consistent with the finding elsewhere using GIST^58^ whose authors rationalized the effect in terms of hydrogen‐bonding groups in the protein being less favorably placed in more constrained environments such as helices and strands. It could also reflect different amino‐acid propensities in different types of secondary structure.

### 3.4 Free energy of water around capped amino acids

**FIGURE 4:**
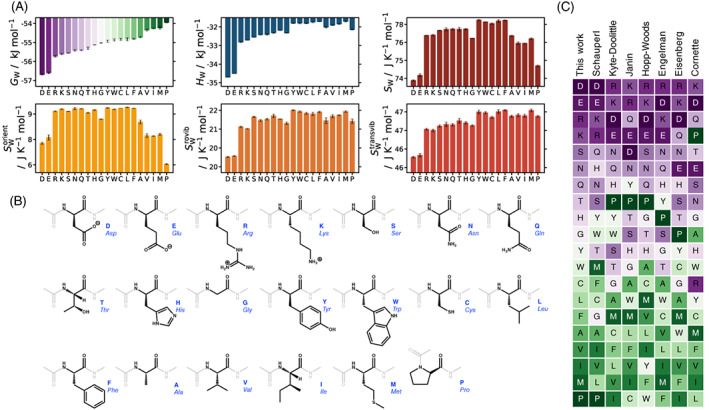
(A) Average free energy $G_{W}$, enthalpy $H_{W}$, and entropy $S_{W}$ per water molecule over all water molecules in the first shell of each capped amino acid, ordered from most stable to least stable free energy, and entropy components of orientational $S_{W}^{\text{or}}$, rotational $S_{W}^{\text{rovib}}$, and translational $S_{W}^{\text{transvib}}$ (bottom left to right). Error bars show the standard deviation for each data point. (B) Structure of each amino acid with capping groups grayed out, ordered from most to least stable hydration shell. (C) Water molecule free energy ordered from most stable/hydrophilic (purple) to least stable/hydrophobic (green) around each amino acid side‐chain calculated here (first column) versus hydrophobicity scales from various other works^121^, ^122^, ^123^, ^124^, ^125^, ^126^

It is insightful to examine the free energy, energy, and entropy of water molecules around single solvated amino acids as a point of comparison with residues in the protein and to compare with a range of widely used hydrophobicity scales for amino acids.^121^, ^122^, ^123^, ^124^, ^125^, ^126^ These values are illustrated in Figure 4(A), ordered from most stable to least stable free energy, together with the water orientational, rovibrational, and transvibrational entropy components. Water molecules closest to capping groups or ions are not included in the analysis. The water energy is most stable around the negative amino acids Asp and Glu due to strong, polar interactions between negatively charged side‐chain oxygens and water‐molecule hydrogen atoms. At the same time, the orientational, rovibrational, and transvibrational entropy components of water are smaller because of the associated stronger forces and torques, partly offsetting the more negative energy. Such EE compensation for water around solutes has been observed in previous studies.^111^, ^112^, ^113^ These effects are present but not as strong for water near positively charged Lys and Arg side chains. This trend of progressively less negative free energy continues as amino acids become more hydrophobic, driven more by energy, less by vibrational entropy and with little dependence on orientational entropy. Only for the most hydrophobic side‐chains does orientational entropy decrease again because of the anisotropic bias of hydrogen donation toward water molecules and away from the hydrophobic solutes. Interestingly, orientational entropy around Pro is most heavily reduced, indicating that water molecules near Pro must have smaller solvation shells, as shown by a low $N_{\mathrm{eff}}$ Equation (9) for water around Pro in Supporting Table S1.

Figure 4(C) makes clear that water free energies in the hydration shell of amino acids show similar trends to those of other hydrophobicity scales, with similar groupings of hydrophobic amino acids at one end and hydrophilic amino acids at the other. One notable point of disagreement between scales is the ranking of Pro, which is most hydrophobic here in agreement with Schauperl's scale^121^ whereas most other scales rank it as more hydrophilic. As noted earlier, this greater hydrophobicity of Pro arises from its low orientational entropy because water molecules near Pro have fewer hydrogen‐bonded neighbors. Interestingly, Leu is ranked as more hydrophilic than Ile, Val, and Ala, even though it is larger than Val and Ala and the same composition as Ile. The dominant contribution to the difference is the orientational entropy, which is markedly higher for Leu and comparable to that of the more polar amino acids, suggesting there are more hydrogen‐bond groups near Leu than for the other three aliphatic amino acids. Also, Met is ranked more hydrophobic here than in most scales, again because of its lower orientational entropy and water coordination number. These effects arising from orientational entropy are subtle and non‐obvious but may be related to the number of hydrocarbon methyl termini of the branched‐chain amino acids which can fit better into water's structure in a clathrate‐like arrangement than bulkier hydrocarbon chains or sulfur atoms of methionine which are more disruptive.

### 3.5 Protein free energy

A breakdown of the entropy, energy, and free energy for each protein is presented in Table 4, including the entropy decomposition according to polymer, residue and UA specified by Equation (1), with the residue and UA terms divided by $N_{R}$ to give per‐residue values.

**TABLE 4:** Protein and residue entropy components, and enthalpy and free energy per residue for each protein

	$S_{P}^{\mathrm{vib}}$	$S_{R}^{\mathrm{vib}}/N_{R}$	$S_{\mathrm{UA}}^{\mathrm{vib}}/N_{R}$	$S_{\mathrm{UA}}^{\text{topo}}/N_{R}$	$S_{\text{prot}}^{\text{total}}/N_{R}$	$H_{\text{prot}}^{\text{total}}/N_{R}$	$G_{\text{prot}}^{\text{total}}/N_{R}$
Protein	($J\;K^{-1}{\mathrm{mol}}^{-1}$)					($\mathrm{kJ}\;{\mathrm{mol}}^{-1}$)	
Lysozyme	91.2	37.9	19.6	3.9	62.1	−71.4	−89.9
α‐Chymotrypsin	87.0	40.9	20.0	4.0	65.3	−12.4	−31.9
α‐Lactalbumin	86.2	37.7	20.7	5.6	64.7	−35.2	−54.5
Ribonuclease Sa	87.2	32.9	17.7	4.0	55.5	−52.6	−69.2

**FIGURE 5:**
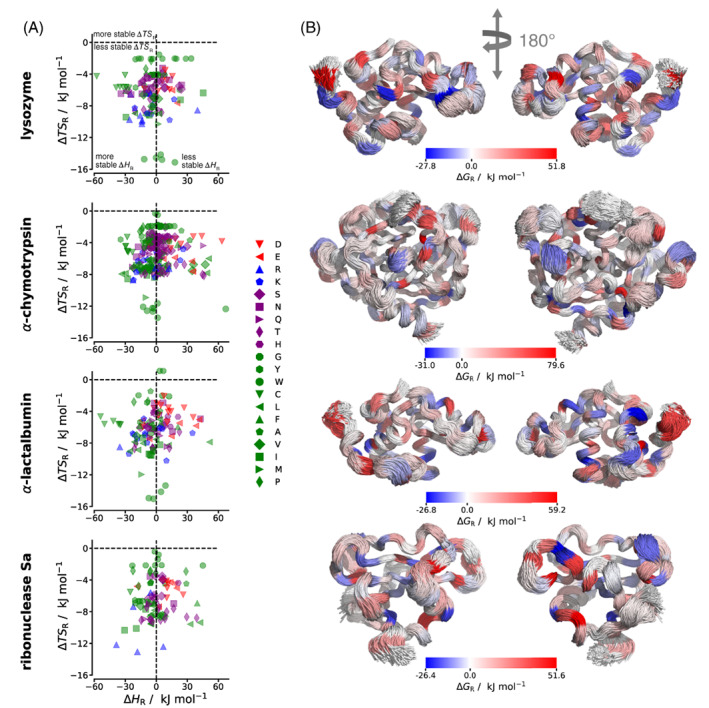
(A) Relative entropy $T\Delta S_{R}$ and energy $\Delta H_{R}$ of residues versus that of single solvated capped amino acids. (B) Per‐residue change in free energy $\Delta G_{R}$ relative to single solvated capped amino acids mapped onto aligned structures from 2500 simulation frames

Similar to before, per‐residue errors in enthalpy were calculated from their standard deviations and found to be in the range 0.01–0.03 kJ mol^−1^, again indicating that the numbers given are accurate to the precision used. Some strikingly large differences are seen for the different proteins but it should be noted that these values depend on the amino‐acid composition of the protein. Lysozyme has the lowest free energy per residue and α‐chymotrypsin the highest, driven mostly by energy but also by entropy. Ribonuclease Sa also has a lower entropy than the other proteins matched by a lower enthalpy. Before interpreting these values, it should be noted that there is a large range of free energies of protein residues (265–638 kJ mol^−1^), as seen by the entropy and enthalpy of single capped solvated amino acids listed in Supporting Table S2. It can be seen that these values depend on a combination of the polarity of the atoms and the size of the amino acids. Evidently, the different kinds of amino acid in each protein mean that absolute values are difficult to compare on the same scale, a problem that does not occur for water molecules earlier, which are all the same. Therefore, to better understand the components of the protein free energies, the relative free energy of each protein residue is analyzed by taking the free energy of the residue and subtracting off the free energy of the corresponding single solvated capped amino acid (Supporting Table S2), excluding the contributions from the capping groups. Terminal residues are not considered in this analysis due to their different numbers of atoms and charges. This referencing is not done for entropy at the protein or residue levels because we do not have reference values for these. Average referenced values $\Delta S_{\mathrm{UA}}$, $\Delta H_{\mathrm{UA}}$, and $\Delta G_{\mathrm{UA}}$ per residue obtained are listed in Table 5 It can be seen that these referenced values in per‐residue form are now much more consistent across all proteins than the absolute values. Moreover, there is a strong enthalpy–entropy compensation in the stability, with negative changes in both enthalpy and entropy bringing about a marginal change in free energy, being stabilizing for lysozyme and α‐chymotrypsin, and neutral for α‐lactalbumin and ribonuclease Sa. The specific referenced values of $T\Delta S_{R}$ and enthalpy $\Delta H_{R}$ of each residue are illustrated in Figure 5(A). When considering relative values, the dependence of relative free energy per residue with local environment is not as clear‐cut as it was for water. The entropy of almost all residues is smaller in proteins than for single capped amino acids, with $T\Delta S_{R}$ values being lower in proteins by up to −15 kJ mol^−1^ (Figure 5(A)). This is expected due to the loss of conformational flexibility brought about by restrained backbone atoms in the polymer but there must also be other vibrational contributions. For energy, the stabilization is much more variable between residues of the same type, varying by up to ±60 kJ mol^−1^. Anionic residues tend to be less stable in proteins, cationic residues are more variable, and, polar and nonpolar residues more often are stabilized but not always. This trend for negatively charged residues is somewhat opposite to water molecules as found earlier, which are stabilized near negative residues. When $\Delta G_{R}$ is mapped onto the 3D protein structures in Figure 5(B), there are no obvious trends, with increases or decreases seemingly randomly spread over the protein surface.

**TABLE 5:** Referenced per‐residue entropy, enthalpy, and free energy for each protein

	$\text{T}\Delta S_{\mathrm{UA}}/N_{R}$	$\Delta H_{\text{prot}}^{\text{total}}/N_{R}$	$\Delta G_{\text{prot}}^{\text{total}}/N_{R}$
Protein	($J\;K^{-1}\;{\mathrm{mol}}^{-1}$)	($\mathrm{kJ}\;{\mathrm{mol}}^{-1}$)	
Lysozyme	−6.4	−8.0	−1.6
α‐Chymotrypsin	−5.3	−6.5	−1.2
α‐Lactalbumin	−6.3	−6.3	0.0
Ribonuclease Sa	−6.2	−6.1	0.1

### 3.6 Correlations between free energy of bonded residues

**FIGURE 6:**
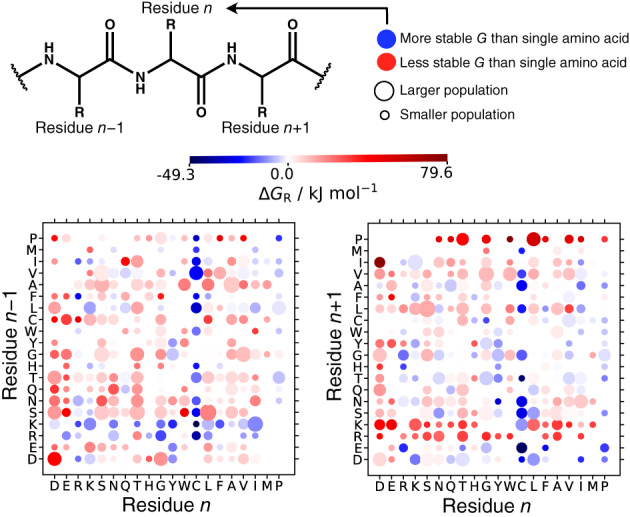
Average relative free energy $\Delta G_{R}$ per residue n over all four proteins when bonded to residue n−1 at the N‐terminal side (left) and residue n+1 at the C‐terminal side (right) of residue n. The number of times a residue pair occurs scales with the size of the dot

To understand what might affect residue free energies, the average relative free energy for each residue type in all four proteins is assessed based on what residue types are bonded to it on its C‐ and N‐terminal sides and plotted in Figure 6. Residues that are more stable than their single solvated amino acid equivalent are shown in blue and less stable residues are shown in red. Further decomposition of energy and entropy is shown in Supporting Figure S4. The trends overall are fairly weak. Most residues are destabilized compared to their single capped reference states, as noted earlier. As might be expected, cystines are more stable regardless of what they are bonded to due to covalent disulfide bonds. Pro, on the other hand, is generally stabilized regardless of the identity of the bonded residues, but if a Pro is on the C‐side of a residue, then it destabilizes that residue. Surprisingly, most residues are stabilized when a positive residue, Arg or Lys, is on the N‐terminal side (n−1) but destabilized if on the C‐terminal side (n+1). For negatively charged Asp and Glu, there is a weaker but reverse trend. The reason for this is not clear but it is likely related to a stabilizing or destabilizing interaction between these charged residues and the backbone amide hydrogen or carbonyl oxygen.

### 3.7 Correlations between water and protein free energy

**FIGURE 7:**
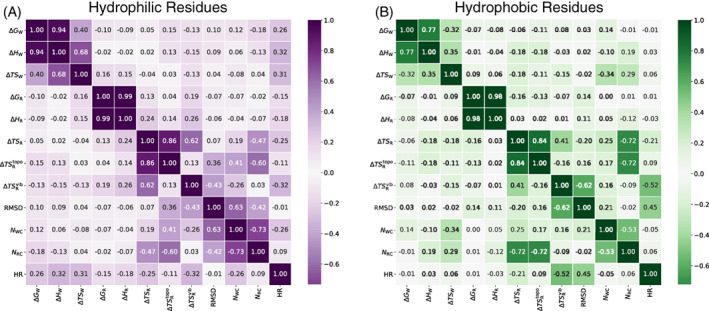
Covariance matrices for thermodynamic and structural properties of (A) hydrophilic residues, namely D, E, R, K, S, N, Q, T, and H and (B) hydrophobic residues, namely G, Y, W, C, L, F, A, V, I, M, and P. Darker shades imply larger correlations

Having examined the free energies of water and protein separately, we finally look for correlations between the free energy values of protein and water. In Figure 7, covariance matrices are presented between free energy, energy, and entropy components together with RMSD, HR, $N_{\mathrm{Wc}}$, and $N_{\mathrm{Rc}}$ (defined in Section 2.7) for hydrophobic and hydrophilic residues separately. As might be expected, residue conformational entropy $\Delta {\mathrm{TS}}_{R}^{\text{topo}}$ is anticorrelated with the number of residue–residue contacts $N_{\mathrm{Rc}}$, more so for hydrophobic residues, because of greater restriction when buried. This effect is also evident in residues with a higher hydrophobic rank HR being more anticorrelated with $\Delta {\mathrm{TS}}_{R}^{\mathrm{vib}}$ because hydrophobic residues are more likely to be buried and so have a lower vibrational entropy due to greater confinement. To further investigate correlations between protein and water quantities, Supporting Figure S5 plots each of $G_{W}$, $N_{\mathrm{Wc}}$, $N_{\mathrm{Rc}}$, or RMSD versus $\Delta G_{R}$ for the corresponding residue of each protein. No obvious trends are seen, apart from hydrophobic residues having surrounding water that is less stable, as has already been noted. Evidently, stability is likely a multi‐body phenomenon that is not easily attributable to the stability of neighboring groups of atoms.

### 3.8 Discussion

Computationally quantifying the thermodynamic properties of proteins in solution is a useful way to understand their behavior in terms of all their constituent parts. Particularly for large, complex systems, it is important to both quantify and decompose free energy in order to go beyond qualitative descriptions such as hydrophobic and hydrophilic to comprehensively understand what atoms and interactions contribute to phenomena such as protein folding, allostery, binding, or catalysis. Having a single consistent framework that addresses all system degrees of freedom that is free from parameters and operates at multiple length scales helps enable a general method for the thermodynamic analysis of such systems. Nevertheless, approximations are still necessary to make EE‐MCC have these properties, such as the choice of a specific hierarchy of structures, the use of the harmonic approximation, which correlations within or between molecules to include, and a number of other issues. The orientational entropy of each protein is not considered here but could be calculated based on its size or coordination number as has been done elsewhere.^79^, ^88^, ^91^, ^127^ This analysis only considers correlations between conformers within a residue and neglects those between residues, which are expected to be small, but should be considered in future work. Another omitted entropy term, the topographical entropy at the residue level, is assumed to be small for stable, relatively rigid proteins as those studied here, but would be larger for systems such as unfolded or intrinsically disordered proteins. The positional entropy arising from mixing with any counterions is not considered here, although in a first approximation one could use the ideal entropy of a mixture. The contributions of water molecules beyond the first shell of the protein have also been ignored because of their near‐bulk‐like properties and to reduce statistical noise. Other studies of hydrated proteins suggest that water contributions to entropy could be included out to 10 Å.^68^

Examining the free energy of water molecules on a per‐residue basis over an ensemble shows the expected trend for the stability of water molecules, being greater adjacent to hydrophilic residues than for hydrophobic residues, as has been reported elsewhere.^47^, ^52^, ^58^, ^60^, ^63^, ^70^, ^77^ Stability is further found to depend on the degree of burial of the water. Fewer water neighbors of a water molecule mostly means lower stability because of its diminished ability to form stabilizing HBs to the surrounding atoms in the more hydrophobic and asymmetric environment that biases orientations and lowers orientational entropy. A third factor affecting stability is having neighboring, negatively charged residues. A larger number of negative residues have previously been found to be suggestive of improved protein solubility.^128^, ^129^ A fourth factor is secondary structure, with helices and strands being surrounded by less stable water.

Probing water molecules is typically the main way to find destabilized regions on a protein to which other molecules might bind. Protein free energy on a per‐residue basis provides a further contribution to understand this process. The thermodynamics of proteins are more difficult to quantify due to their large size, flexible nature, and correlated motions. The multiscale formulation employed here with three levels of hierarchy, polymer, monomer, and UA length scales, enables protein and water entropy to be better understood in terms of multiple correlating units. We do not observe any significant correlations between protein free energy and surrounding water molecules. This may simply reflect the expectation that the free energy of protein residues is dominated by their surrounding residues, whether bonded or nonbonded, because of the strong covalent interactions for the bonded ones and close‐packing of the others, whereas the free energy of water is governed by the atoms that their neighboring residues present to them. However, using single‐amino acid reference states, we are able to detect intriguing stabilization for lysozyme and α‐chymotrypsin not seen for α‐lactalbumin and ribonuclease Sa.

Despite this lack of correlation, we do make some intriguing findings. By assessing each residue based on what is bonded to its N‐ and C‐terminal sides, greater residue stabilization is found when a cationic residue is bonded to the N‐side, compared to destabilization when the cationic residue is on the C‐side. The opposite trend is observed, albeit slightly weaker, for anionic residues. These asymmetric distributions of free energy changes may be due to how charged residues interact with atoms on the protein backbone. We hypothesize that cationic residues interact more favorably with the backbone carbonyl group on the next residue in the sequence. Conversely, anionic residues interact more favorably with the backbone amide group on the previous residue in the sequence. These asymmetric side‐chain backbone interactions may cause a strain in the backbone on the opposite side of the direction in which the interaction takes place, resulting in instability. Further analysis of UA interactions between residues may highlight these side‐chain backbone interactions. A similar asymmetric observation has been shown in other work of QM calculations that the αC–C′ backbone bond length is reduced when an anionic residue is in the n+1 position due to higher electron density, whereas a basic residue in the same position reduces the bond length.^130^

### 3.9 Conclusion

We present an EE method to calculate the free energy, energy, and entropy of a hydrated protein from MD simulations and apply it to individual hydrated amino acids and to four globular proteins, namely lysozyme, α‐chymotrypsin, α‐lactalbumin, and ribonuclease Sa. Entropy and energy are calculated for sets of atoms over a hierarchy of length scales from UAs to residues to whole molecules. The decomposition of free energy of water molecules based on their neighboring residues gives detailed information about how protein interactions influence neighboring water molecules. Free energy decomposition also allows for the study of the interplay between the stability of water and their neighboring residues. Strong correlations were not observed between water and residue stability, although we did detect stabilization of water next to anionic residues relative to cationic residues and of water next to loops relative to that near helices and strands. Another intriguing finding was the discovery of an asymmetry in the stability of residues depending on whether charged residues were on their N or C‐terminal sides. EE‐MCC with its insightful decomposition of free energy over groups of atoms, its hierarchy of length scales, and its single consistent formulation over all atoms in the system, should be readily scalable to larger and more flexible systems, such as protein–ligand complexes, protein assemblies, and intrinsically disordered proteins, as well as many other kinds of molecular systems.

## Supporting information


**APPENDIX S1** Supplementary Information

## Data Availability

The methods used to generate the data in this study are openly available in PoseidonBeta at https://github.com/jkalayan/PoseidonBeta and CodeEntropy at https://github.com/arghya90/CodeEntropy.
