# Supplementary material for: Total free energy analysis of fully hydrated proteins
Source: Proteins. 2022 Aug 25;91(1):74–90. doi: 10.1002/prot.26411 (PMC10087023; doi:10.1002/prot.26411)
Supplement: Supplementary file 1 — APPENDIX S1 Supplementary Information [file PROT-91-74-s001.pdf]

Supporting Information for "Total Free Energy Analysis of Fully Hydrated Proteins"

Jas Kalayan,[*,†] Arghya Chakravorty,[‡] Jim Warwicker,[†] and Richard H. Henchman[¶]

†*Manchester Institute of Biotechnology, The University of Manchester, 131 Princess Street, Manchester M1 7DN, United Kingdom and School of Chemistry, The University of Manchester, Oxford Road, Manchester M13 9PL, United Kingdom*

‡*Department of Chemistry and Biophysics, University of Michigan, 930 N University Ave, Ann Arbor, MI 48109*

¶*Sydney Medical School, Faculty of Medicine and Health, University of Sydney, Camperdown, NSW, 2006, Australia*

E-mail: jas.kalayan@manchester.ac.uk

Table S1: Components of Orientational Entropy for Water Molecules around Amino Acids

Amino acid	N_c	N_{eff}	$p(HB)_{av}$	S_W^{or} (J/K/mol)
D/ Asp	6.94	5.44	0.1989	6.93 (0.05)
E/ Glu	6.93	5.45	0.1990	6.97 (0.11)
R/ Arg	7.12	6.06	0.2155	8.80 (0.02)
K/ Lys	7.08	6.11	0.2183	8.94 (0.02)
S/ Ser	7.01	6.03	0.2178	8.76 (0.01)
N/ Asn	7.00	6.11	0.2207	9.04 (0.05)
Q/ Gln	6.99	6.10	0.2209	9.03 (0.01)
T/ Thr	6.99	5.99	0.2174	8.74 (0.02)
H/ His	7.04	6.08	0.2185	8.93 (0.02)
G/ Gly	7.00	5.91	0.2143	8.36 (0.01)
Y/ Tyr	7.01	6.11	0.2208	9.08 (0.02)
W/ Trp	7.05	6.06	0.2180	8.94 (0.02)
C/ Cys	7.03	6.12	0.2204	9.02 (0.00)
L/ Leu	6.91	6.11	0.2232	9.09 (0.01)
F/ Phe	7.02	6.09	0.2201	9.04 (0.01)
A/ Ala	6.91	5.80	0.2130	8.10 (0.08)
V/ Val	6.94	5.61	0.2042	7.38 (0.09)
I/ Ile	6.94	5.57	0.2029	7.24 (0.02)
M/ Met	7.00	5.62	0.2033	7.38 (0.05)
P/ Pro	6.98	5.25	0.1888	5.99 (0.02)

[a] Number of water molecules in the amino acid coordination shell N_c
[b] Effective number of neighbours N_{eff} available for hydrogen-bonding
[c] Bias in HBs $p(HB_{av})$, where 0.25 equals no bias in donating and accepting
[d] Water orientational entropy S_W^{or}
[e] Standard errors (SE) in parentheses of S_W^{or} over $n = 3$ repeated simulations, SE=$\sigma/\sqrt{n-1}$ and σ is the standard deviation

Table S2: Entropy, Enthalpy and Free Energy of Single Capped Solvated Amino Acids[a]

Amino acid	TS (kJ/mol)	H (kJ/mol)	G (kJ/mol)
D/ Asp	11.5 (0.4)	-242.5 (0.2)	-254.0 (0.3)
E/ Glu	16.9 (0.1)	-210.0 (0.2)	-226.9 (0.2)
R/ Arg	23.5 (0.5)	-614.7 (0.4)	-638.2 (0.9)
K/ Lys	20.2 (0.4)	55.3 (0.0)	35.0 (0.3)
S/ Ser	9.2 (0.3)	28.5 (0.5)	19.3 (0.2)
N/ Asn	12.6 (0.2)	-252.5 (0.1)	-265.1 (0.2)
Q/ Gln	16.5 (0.5)	-175.4 (0.2)	-191.9 (0.5)
T/ Thr	14.0 (0.4)	-30.6 (0.2)	-44.5 (0.6)
H/ His	13.5 (0.9)	97.7 (0.4)	84.2 (0.5)
G/ Gly	2.8 (0.1)	6.4 (0.1)	3.6 (0.2)
Y/ Tyr	16.4 (0.4)	32.3 (0.3)	15.9 (0.5)
W/ Trp	26.8 (0.9)	156.0 (0.3)	129.2 (1.1)
C/ Cys	9.6 (0.4)	77.8 (0.1)	68.2 (0.4)
L/ Leu	15.9 (0.8)	27.9 (0.4)	12.0 (0.4)
F/ Phe	14.3 (0.4)	116.1 (0.3)	101.7 (0.4)
A/ Ala	6.0 (0.3)	58.9 (0.0)	52.9 (0.4)
V/ Val	13.3 (0.3)	20.8 (0.3)	7.5 (0.2)
I/ Ile	16.6 (0.7)	117.2 (0.2)	100.7 (0.5)
M/ Met	20.5 (0.1)	94.1 (0.3)	73.6 (0.3)
P/ Pro	8.0 (0.5)	116.6 (0.2)	108.6 (0.5)

[a] Standard errors (SE) in parentheses over $n = 3$ repeated simulations, SE$=\sigma/\sqrt{n-1}$ and σ is the standard deviation

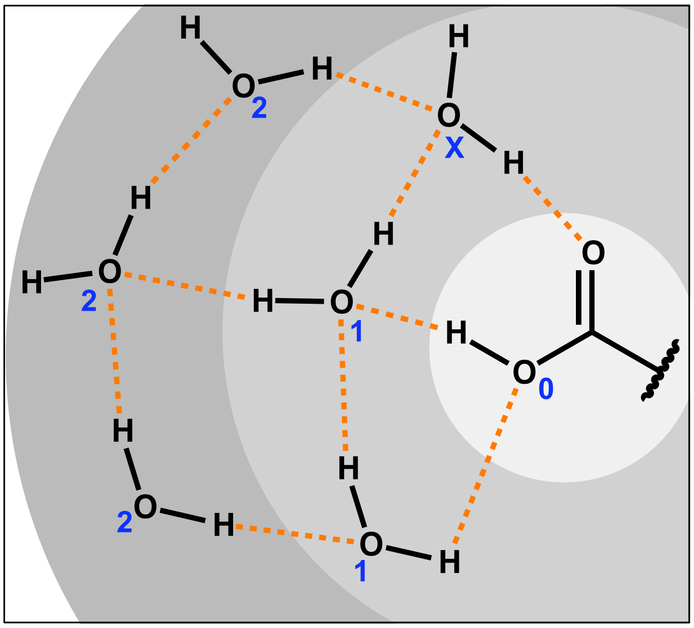

Figure S1: Schematic representation of how neighbours are labelled for water molecule orientation statistical analysis. Each water in the central water coordination shell is defined by the hydration shell it is in with respect to the nearest solute. Water molecules in the first shell of the solute are labelled as "1" unless a water is closer to another united atom on the solute, then the water is labelled as "X". All water molecules beyond the first shell are labelled as "2". The central water thus has neighbours ordered alphanumerically as "0,1,2,2,2,X" where "0" refers to the closest solute united-atom. If applicable, any further solute united-atoms in a coordination shell are labelled as the heavy atom name. The central water in this example donates to neighbours "2,X" and accepts from neighbours "0,1".

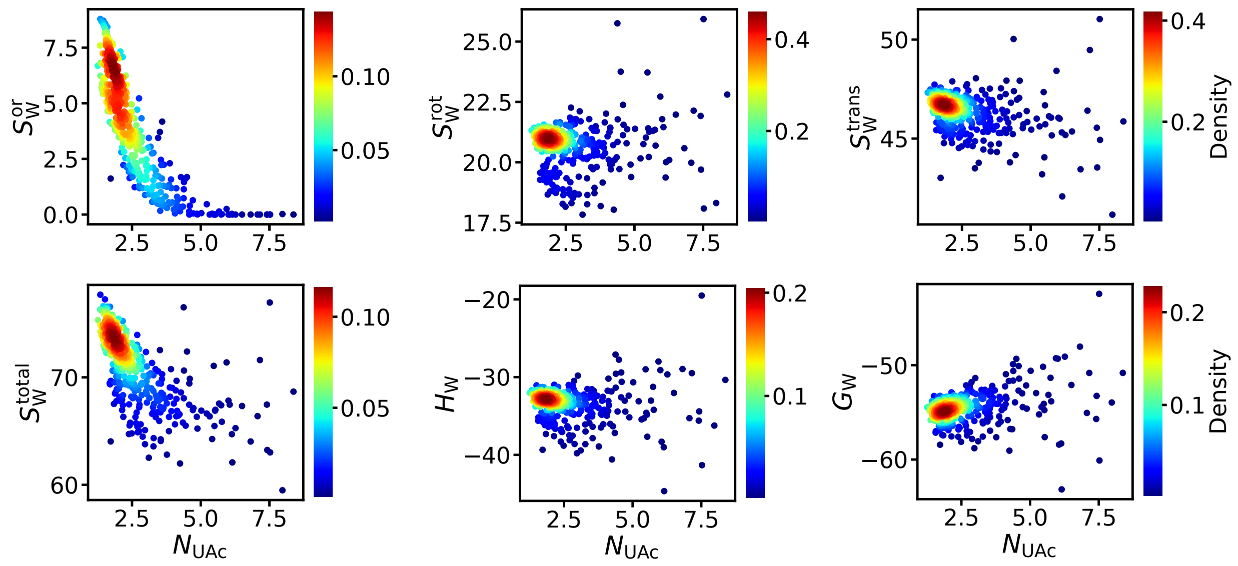

Figure S2: Effect of the number of surrounding protein united atoms, N_{UAc}, on water entropy and its orientational, rotational and translational components (J K^{-1} mol^{-1}), and on water enthalpy and free energy (kJ mol^{-1}). The colours represent the density of the points and each point represents water molecules closest to a particular residue.

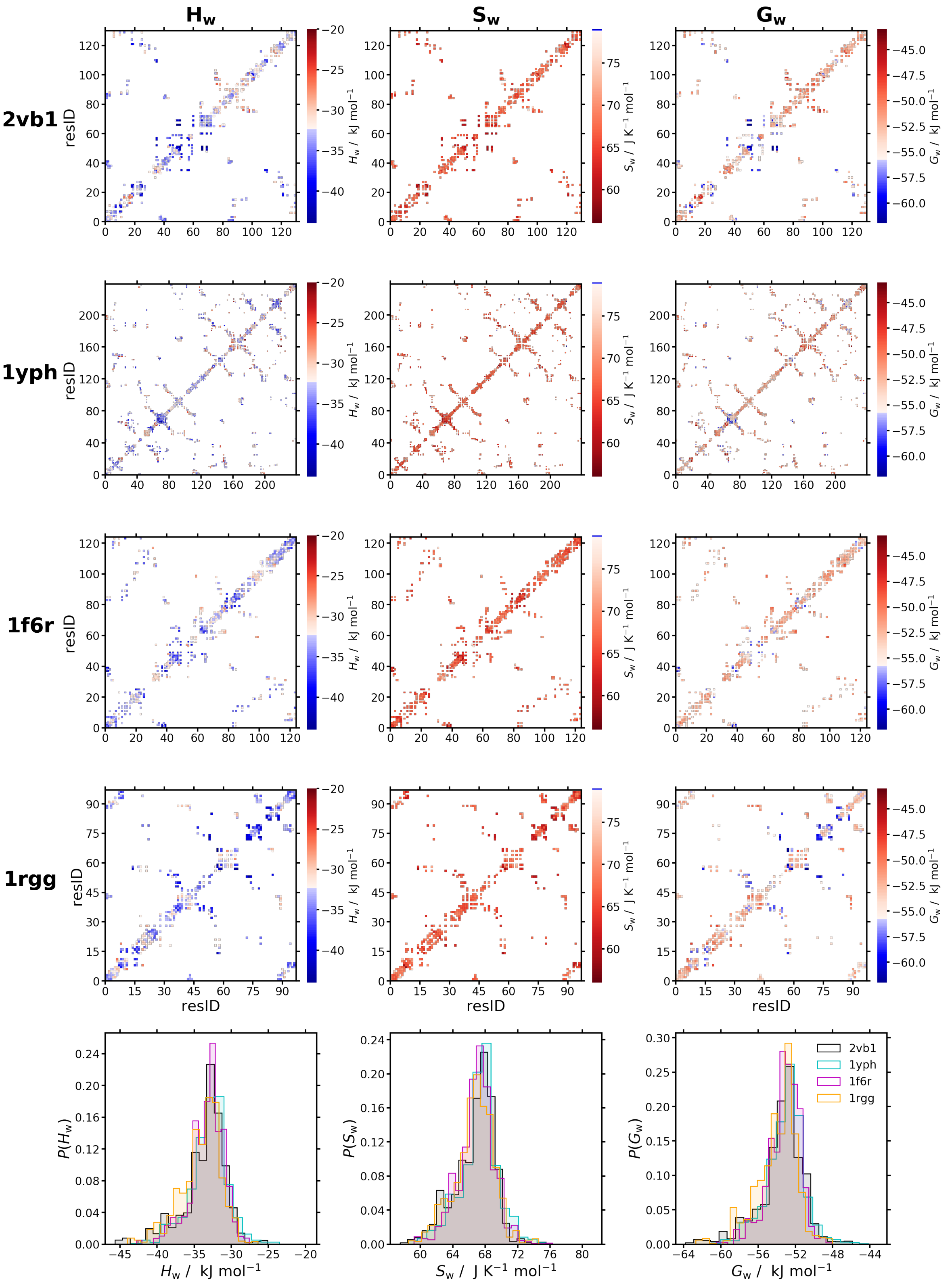

Figure S3: Enthalpy H_W, entropy S_W and free energy G_W of water molecules in contact with at least two protein residues for the lysozyme (2vb1), α-chymotrypsin (1yph), α-lactalbumin (1f6r) and ribonuclease Sa (1rgg). Points are coloured red for less stable and blue for more stable than bulk water. The bottom row displays histograms for the same three quantities, coloured according to the protein.

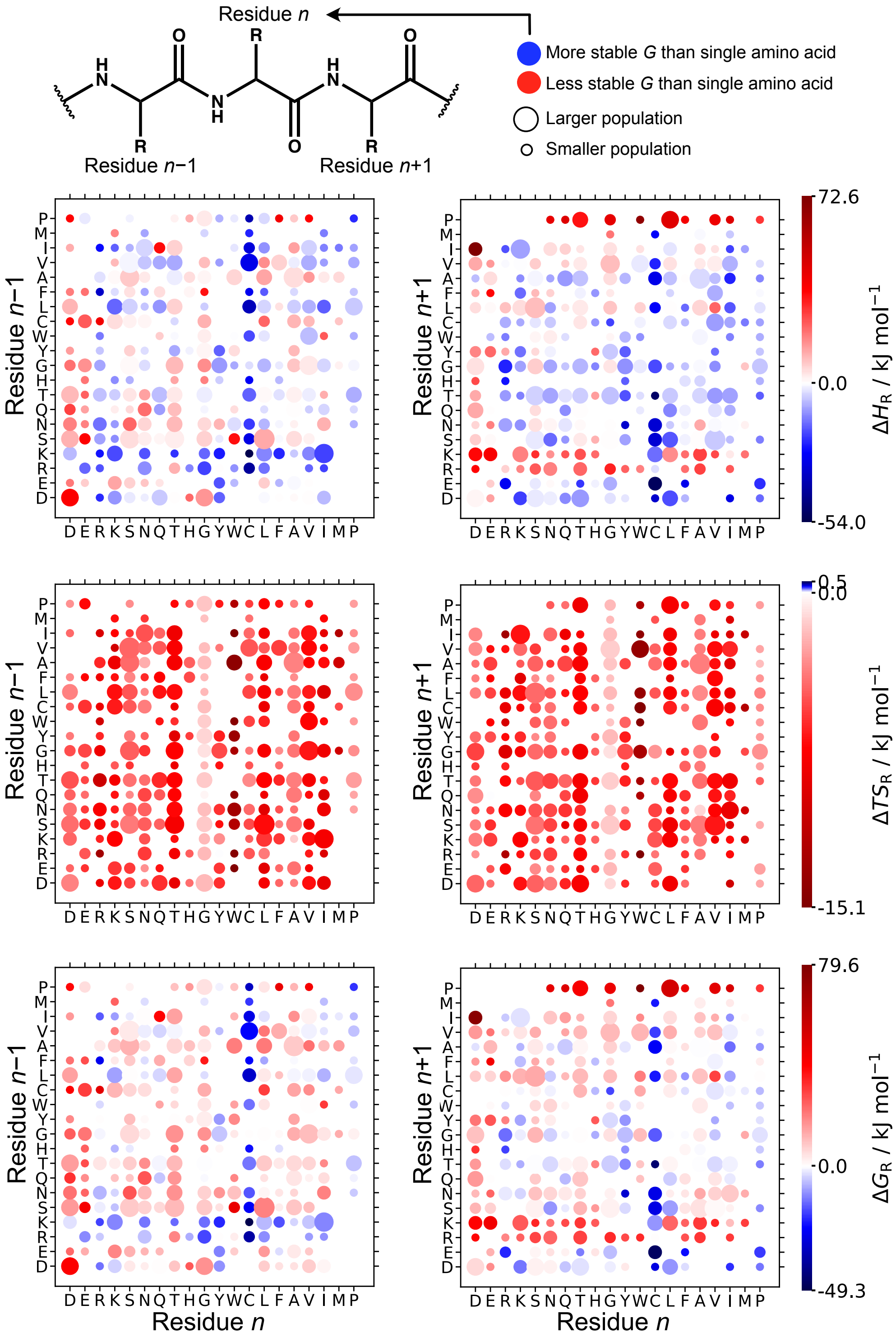

Figure S4: From top to bottom, the average relative enthalpy ΔH_{R}, entropy ΔTS_{R} and free energy ΔG_{R} of each residue type in the n position depending on what residue is in the $n-1$ position (first column) and in the $n+1$ position (second column).

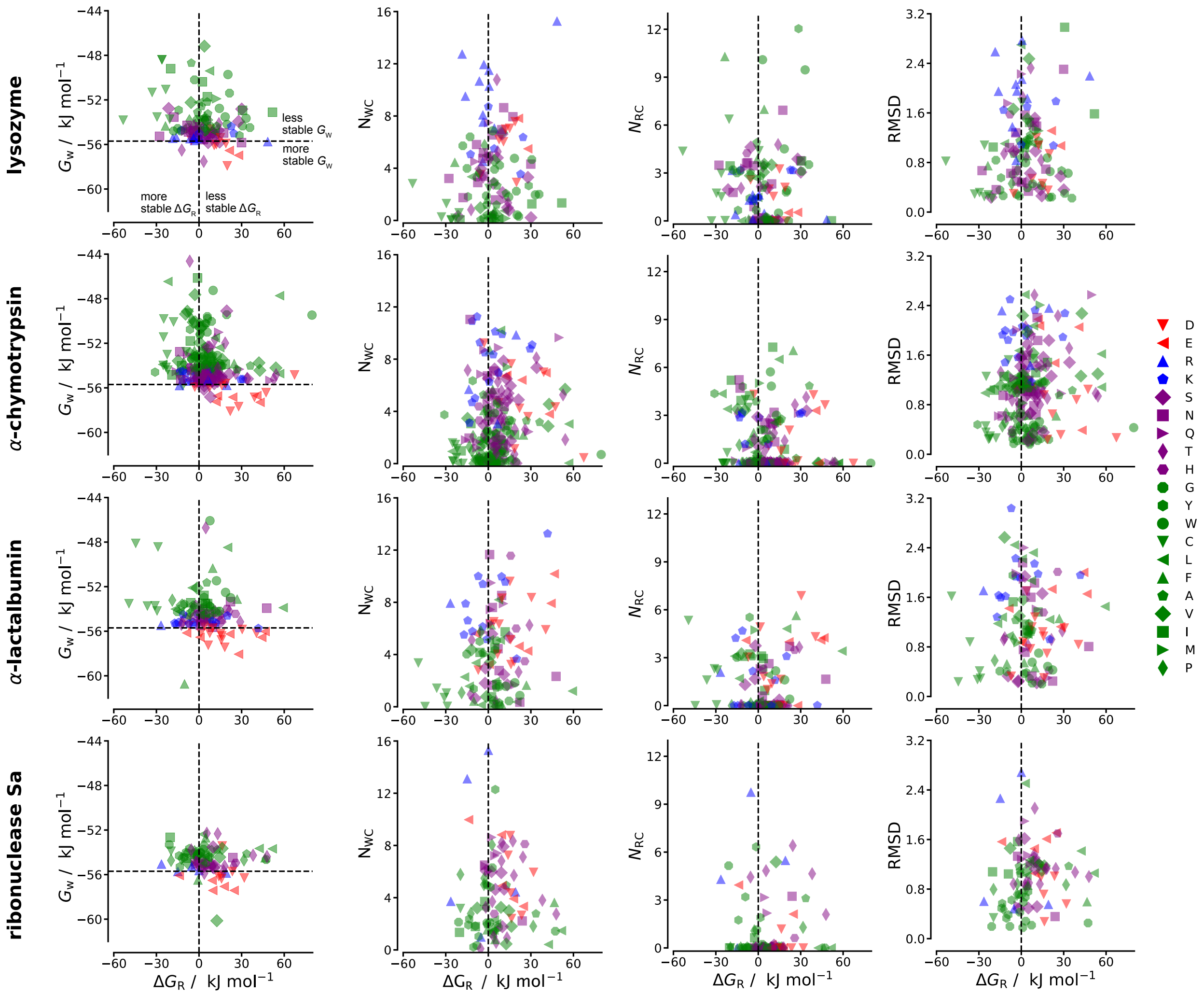

Figure S5: Free energy change of each residue in each protein ΔG_{R} is plotted on the x-axis and (from left to right) free energy of surrounding water molecules G_{W}, number of water molecules in a residue coordination shell N_{WC}, number of contacts with neighbouring residues N_{RC} and RMSD per residue on y-axes.
